# Cancer stem cells in progression of colorectal cancer

**DOI:** 10.18632/oncotarget.23607

**Published:** 2017-12-22

**Authors:** Yujuan Zhou, Longzheng Xia, Heran Wang, Linda Oyang, Min Su, Qiang Liu, Jingguan Lin, Shiming Tan, Yutong Tian, Qianjin Liao, Deliang Cao

**Affiliations:** ^1^ Hunan Key Laboratory of Translational Radiation Oncology, Hunan Cancer Hospital and the Affiliated Cancer Hospital of Xiangya School of Medicine, Central South University, Changsha, 410013, Hunan, China; ^2^ Department of Medical Microbiology, Immunology & Cell Biology, Simmons Cancer Institute, Southern Illinois University School of Medicine, Springfield, IL, 62794, USA

**Keywords:** colorectal cancer, cancer stem cells, metastasis, epithelial mesenchymal transition, tumor microenvironment

## Abstract

Colorectal cancer is one of the most common cancers worldwide with high mortality. Distant metastasis and relapse are major causes of patient death. Cancer stem cells (CSCs) play a critical role in the metastasis and relapse of colorectal cancer. CSCs are a subpopulation of cancer cells with unique properties of self-renewal, infinite division and multi-directional differentiation potential. Colorectal CSCs are defined with a group of cell surface markers, such as CD44, CD133, CD24, EpCAM, LGR5 and ALDH. They are highly tumorigenic, chemoresistant and radioresistant and thus are critical in the metastasis and recurrence of colorectal cancer and disease-free survival. This review article updates the colorectal CSCs with a focus on their role in tumor initiation, progression, drug resistance and tumor relapse.

## INTRODUCTION

Advance in diagnosis and treatment of colorectal cancer (CRC) is not well translated into a favorable clinical outcome, and the disease-free survival of CRC remains poor [1]. A critical challenge in management of colorectal cancer is metastasis and relapse of the disease [2].

Cancer stem cells (CSCs) are a group of tumor cells with stem cell characteristics of self-renewal, infinite proliferation, and potential of multi-directional differentiation [3, 4]. The CSCs account for a very minor population of cancer, but are closely related to tumor metastasis, drug resistance and recurrence after primary treatment [5]. Traditional drugs and radiotherapy may make solid tumors relieved, but not kill CSCs. These CSCs may enter dormant status after treatment intervention and then become a source of cancer recurrence [6]. To date, CSCs are a great concern in treatment response and prognosis of various cancers, including colorectal cancer.

## COLORECTAL CANCER STEM CELLS

CSCs were described first in acute myeloid leukemia (AML), but soon evidenced in solid tumors. The CSCs arise by gene mutations or deregulation of genetic programs in normal stem/progenitor cells [7]. In CRC, cells with epithelial characteristics, i.e., EpCAM ^high^/CD44^+^ are isolated and these cells show stem cell-like properties, such as tumorigenesis, invasion and metastasis, indicating the epithelial source of CSCs [8]. In general, colorectal CSCs are mainly derived from differentiated intestinal cells or intestinal stem cells (ISCs) through gains of genetic alterations that are sufficient to induce malignant transformation [9].

Colorectal CSCs share the major biological characteristics of stem cells from other solid tumors [6], including 1) self-renewal and multi-directional differentiation potential, 2) abnormal activation of proliferating signaling pathways, such as Wnt, Notch and Hedgehog, 3) high tumorigenicity, and 4) strong drug and/or radiation resistance [10]. The colorectal CSCs also share many features of normal intestinal stem cells, such as infinite division, telomerase activity and organ-specific differentiation [11].

**Figure 1:**
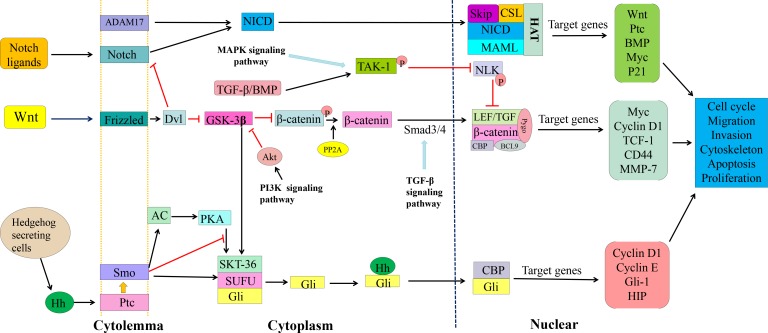
Wnt/β-catenin, Notch and Hedgehog signaling pathways and cross-talks in CSC cells Wnt/β-catenin signaling is transmitted through Frizzled (FZD) receptor and inhibits disheveled (Dvl) and then glycogen synthase kinase-3β (GSK-3β), thereby stabilizing β–catenin. Accumulated β–catenin translocates into the nucleus where it binds to TCF/LEF transcription factors and form a complex of TCF/LEF/β-catenin/Pygo/CBP/BCL-9, regulating expression of target genes. Wnt/β-catenin signaling cross-talks with Notch, MAPK and TGF-β signaling. In Notch signaling, binding of Notch ligands to the receptor results in two proteolytic cleavages to release NICD. The released NICD then translocates into the nucleus where it interacts with the transcription factors, forming the complex of Skip/CSL/NICD/MAML/HAT to activate expression of Wnt, Ptc, BMP, Myc, and P21. Notch signaling cross-talks with the PI3K cascade. In Hedgehog signaling, Hh ligand secreted by Hedgehog secretory cells binds to PTCH1 (Ptc) and generates activated Gli/CBP that translocates into the nucleus and induce the expression of target genes, such as Cyclin D1, Cyclin E, Gli-1 and HIP. These main survival pathways and cross-talks among themselves and with other signaling pathways, i.e., TGF-β, MAPK and PI3K constitute a complex regulatory network for survival and proliferation of cancer stem cells.

Cancer stem cells preferentially demonstrate persistent activation of multiple signal transduction pathways for stemness maintenance and self-renewal [12, 13]. The abnormal signaling pathways that have been well addressed in colorectal CSCs, including Wnt/β-catenin, Notch, TGF-β and Hedgehog (Figure 1) [14–16]. The Wnt/β-catenin pathway is particularly important in stemness maintenance and drug resistance of colorectal CSCs [17]. Wnt/β-catenin signaling is activated through binding of Wnt ligands with Frizzled receptor complex, and β-catenin, a transcription co-regulator, is a key effector of this Wnt/β-catenin signaling [18–20]. Whether Wnt/β-catenin signaling functions depends on the level and cellular location of β-catenin, and GSK-3β, a multifunctional kinase located in the regulatory APC/Axin/GSK-3β complex, is a negative regulator of β-catenin. This GSK-3β phosphorylates β-catenin and drives ubiquitination and proteasomal degradation through the β-TrCP/Skp pathway. Activation of the Wnt/receptor complex displaces GSK-3β from APC/Axin and thus stabilizes β-catenin. Accumulated β-catenin translocates into the nucleus, binds to LEF/TCF transcription factors, and drives target gene expression. In colorectal CSCs, the mutations that prevent formation of the APC/Axin/GSK-3β destruction complex lead to accumulation and nuclear translocation of β-catenin, activating the target genes involved in stemness and differentiation [21].

Colorectal CSCs are identified via a group of surface markers. The main colorectal CSC markers documented are CD44, CD133, CD166, Lgr5, ALDH1 and EpCAM [8, 22]. Other more universal CSC markers include Nanog, Sox2, Oct-4, CD51, CD24, CD26 and CD29 [8, 22–29]. In addition to being a surface marker, these molecules are biologically functional. Table 1 summarizes the colorectal CSC markers and their cellular function identified thus far. Introduced below are several important colorectal CSC markers.

**Table 1:** Surface markers of colorectal cancer stem cells

Name of Marker	Function	References
CD44	Cell surface glycoprotein involved in malignant progression, cell adhesion and migration, less sensitive to apoptosis signals and more resistance to therapies	[30, 31], [114]
CD24	Cell adhesion	[27], [115]
CD133	Cell transmembrane glycoprotein involved in regulation of stemness, associated with cancer local recurrence and survival	[8], [32–35], [116]
CD166	Cell adhesion molecule involved in neuronal extension, embryonic hematopoiesis and embryonic angiogenesis, associated with the development of adenoma to carcinoma	[8], [40], [39]
Lgr-5	Expressed in intestinal stem cells and a downstream target of Wnt pathway, related to tumorigenesis, 5-fluorouracil resistance and recurrence of colorectal cancer	[41–42]
EpCAM	Cell adhesion molecule involved in Cadherin-Catenin and Wnt pathway, associated to lymph node metastasis, vascular invasion and distant metastasis	[50–52]
ALDH1	Associated with poor differentiation, metastasis and drug resistance, correlated with advanced-stage of colorectal cancer	[47], [49]
Oct 4	Regulation of stemness, negatively correlated with cancer death, lymphatic invasion and lymph node metastasis	[117, 118]
SOX-2	Regulation of stemness, correlated with recurrence and lower disease-free survival	[118]
Nanog	Transcriptional regulator, self-renewal	[117], [119]
CD29	Cell adhesion molecule regulating differentiation and self-renewal, involved in embryogenesis, hemostasis, tissue repair, immune response and cancer metastases	[29], [120–122]
CD26	Cell adhesion molecule involved in migration and invasion	[28]
CD51	Associated with the EMT of colorectal cancer cells, sphere formation, cell motility and tumor formation	[26]

### CD44

CD44 is a hyaluronic acid receptor encoded by CD44 gene. It is a transmembrane glycoprotein that regulates cell-cell interaction, cell adhesion and migration [30]. CD44^+^ colon cancer cells display aggressive proliferation, high colony formation, insensitivity to apoptosis and resistance to chemo- and radiotherapies when compared to CD44 negative cells [22]. Knockdown of CD44 with short hairpin RNA (shRNA) leads to decrease of cell proliferation, migration and invasion, but to inhibition of apoptosis [31]. In the CD44-silenced HCT116 colon cancer cells, Bax was increased while Bcl-2 and Bcl-xL were downregulated, leading to cleavages of caspase-3, caspase-9 and PARP [31]. Therefore, this CD44 marker may be a potential therapeutic target of colorectal cancer.

### CD133

CD133 (also named AC133 or prominin-1) is a transmembrane glycoprotein expressed in hematopoietic cells, endothelial cells and neuroepithelial cells [32]. CD133^+^ colorectal cancer cells show stem cell characteristics, such as self-renewal and multi-directional differentiation potential [8]. CD133 is considered a specific marker of primary colorectal CSCs and the CD133 expression is associated with colorectal cancer cell differentiation and tumor size [33]. CD133^+^ colorectal cancer cells are also resistant to radio- and chemotherapy [34, 35]. However, controversial results were reported, where the CD133^-^ cells may be more aggressive [36, 37].

### CD166

CD166 is a adhesion molecule of leucocytes, participating in intercellular interaction and cell adhesion with extracellular matrix [38]. In colorectal cancer, CD166 expression is correlated with the disease pathogenesis, being an early event in colon carcinogenesis [39]. However, CD166 expression was associated with a smaller size of the primary tumor in a meta-analysis [40]. As a CSC marker, therefore, CD166 needs to be considered together with other markers, such as CD44, CD24, CD29 and CD26 [8].

### Lgr5

Leucine-rich repeat-containing G protein-coupled receptor 5 (Lgr5) belongs to the family of G protein-coupled receptors, which contains 17 leucine-rich repeats and a transmembrane domain of α-helix. Lgr-5 is a marker of normal intestinal stem cells [41] and also colorectal CSCs [42]. Lgr-5 plays an active role in pathogenesis of colorectal cancer [43], and the Lgr5 expression is closely related to tumorigenesis, 5-fluorouracil resistance and recurrence of colorectal cancer [43, 44]. In stage IV colorectal cancer, high Lgr5 expression is associated with poor prognosis [45]. Therefore, Lgr5 is considered an indicator of poor prognosis and a potential target of colorectal cancer.

### ALDH1

Aldehyde dehydrogenase isoform 1 (ALDH1) catalyzes conversion of aldehyde to carboxylic acid. ALDH1 is often used as a surrogate marker of CSCs and non-CSCs in different cancers [46]. In colorectal cancer, ALDH1 serves as a CSC marker, and a high level of ALDH1 is associated with poor differentiation and high metastasis. ALDH1 is also a mediator of drug resistance in colorectal CSCs [47], and in rectal cancer, preoperative radiochemotherapy induces expression of ALDH1 [48]. However, opposite observations were reported in which loss of ALDH1 expression was correlated with advanced stage of colorectal cancer [49]. Further study may be needed to define its role in development and progression of colorectal cancer.

### EpCAM

EpCAM is a 40 kDa single transmembrane protein encoded by tumor-associated calcium signal transducer-1 (TACSTD-1) gene [50]. EpCAM is involved in regulation of intercellular adhesion-mediated signaling transduction, cell migration, proliferation and differentiation [50]. As an epithelial adhesion molecule, EpCAM plays a role in carcinogenesis of epithelial cells by activating expression of proto-oncogenes c-myc and cyclin A/E [51]. In addition, EpCAM blocks antigen presentation of dendritic cells, driving escaping of the CD4^+^ T-cell dependent immune surveillance [52]. EpCAM can also potentiate the canonical WNT/β-catenin signaling cascade through intra-membrane proteolysis and subsequent nuclear translocation of its intracellular C-terminal domain, leading to cross-talk with the Notch, Hedgehog and TGFβ/BMP signaling cascades [53]. This constitutes the stem cell signaling network and regulates expression of other functional CSC markers [54–56]

## COLORECTAL CSCS IN PROGRESSION OF COLORECTAL CANCER

CSC markers function in cell proliferation, metastasis and radio-/chemoresistance; and multiple cell proliferating cascades are activated in CSCs. Therefore, CSCs are critical to cancer progression and prognosis.

### Colorectal CSCs and cancer metastasis

Distant metastasis of cancer cells is a complex process, including shedding and invading of primary cancer cells into circulatory system, migration and penetration of the vessel endothelial cell layer, tissue invasion, cell proliferation and angiogenesis [57]. Colorectal CSCs are “seed” cells for invasion and metastasis of colorectal cancer to distant organs, due to 1) infinite division, 2) plasticity to better adapt a new external microenvironment different from the primary tumor site, and 3) heterogeneity derived from asymmetric division, which produces a variety of heterogeneous tumor cells for the new microenvironmental selection. This also explains the heterogeneity of tumor cells in metastatic masses.

### Migrating cancer stem cells and colorectal cancer metastasis

Not all CSCs in primary lesions are metastatic, and metastatic tumors are produced from a specific subpopulation of CSCs, named migrating cancer stem cells (MCSCs) [58, 59]. Brabletz's and Oskarsson's groups classified colorectal CSCs into two subgroups, i.e., stationary cancer stem cells (SCSCs) and MCSCs [60, 61]. The SCSCs exist in the epithelial tissues and are active even in benign precursor lesions. SCSCs mainly contribute to proliferation of the tumor mass *in situ* and remain in the differentiated area throughout tumor progression; they cannot disseminate [60]. The MCSCs lead to the rapidly invasive growth and dissemination of tumor cells [62]. MCSCs divide asymmetrically and generate differentiating cancer cells to start new proliferation and differentiation locally; the MCSCs then migrate a short distance and undergo a new asymmetric division to enlarge the primary tumor [63, 64]. SCSCs and other tumor cells can be transited into MCSCs in primary or metastatic tumor mass [65].

More interestingly, organ-specific metastases of cancer may be initiated by different MCSCs that have organ-unique characteristics. For example, CD110^+^ colorectal MCSCs are prone to colorectal-liver metastases (CRLM), but the colorectal MCSCs with a high level of CDCP1 are easier to colorectal-pulmonary metastases (CRPM) [11]. Nevertheless, specific surface markers of MCSCs are still under identification and further efforts are needed to accurately distinguish MCSCs and SCSCs. Furthermore, the CSCs may gradually evolve into MCSCs through epithelial mesenchymal transition (EMT) after formation of metastatic foci in distant organs [66].

### EMT, CSCs and metastasis of colorectal cancer cells

Epithelial mesenchymal transition (EMT) is characterized by loss of epithelial morphology and markers but gains of mesenchymal features and markers. EMT is a basic process of organ development during the embryonic development [67]. Cancer cells that undergo EMT acquire stemness [68]. Indeed, non-CSCs acquire CSC-like characteristics, capacity of seeding tumors and surface markers through EMT [69]. The colorectal cancer cells that undergo EMT exhibit properties of EMT and CSCs, such as high expression of Snail, Lgr5, CD133, CD44 and EpCAM [70–73].

Signaling pathways involved in EMT, e.g., TGF-β, Wnt and Notch, also play roles in CSCs [74–76]. For instance, TGF-β1 induces expression of EMT markers (such as Slug, Twist1, β-catenin and N-cadherin) and also upregulates CSC markers (e.g., Oct4, Sox2, Nanog and Klf4) in colorectal cancer. Snail and Nanog signaling promotes EMT and acquisition of stemness in colorectal cancer cells, such as self-renewal, tumorigenicity, metastasis and drug resistance [77, 78]. The colorectal cancer cells with a high level of Nanog show stem cell properties and high expression of Slug, a driver of EMT through the IGF/STAT3/NANOG/Slug cascade.

CSCs and EMT processes interact at molecular levels [70]. CSC marker CD51 is co-localized with type I TGF-β receptor (TβRI) and type II TGF-β receptor (TβRII) and enhances the TGF-β dependent accumulation of p-Smad2/3 in the nucleus, which upregulates EMT-related genes, such as PAI1, MMP9 and Snail, and promotes sphere formation, cell motility and tumor formation [26]. Therefore, it is speculated that metastasis of colorectal cancer is due to the EMT of colorectal CSCs, leading to loss of epithelial characteristics and acquisition of mesenchymal phenotypes. This process offers colorectal CSCs the ability of migration and invasion through degradation of extracellular matrix and infiltration into distant organs [79].

### Tumor microenvironment, colorectal CSCs and cancer metastasis

**Figure 2:**
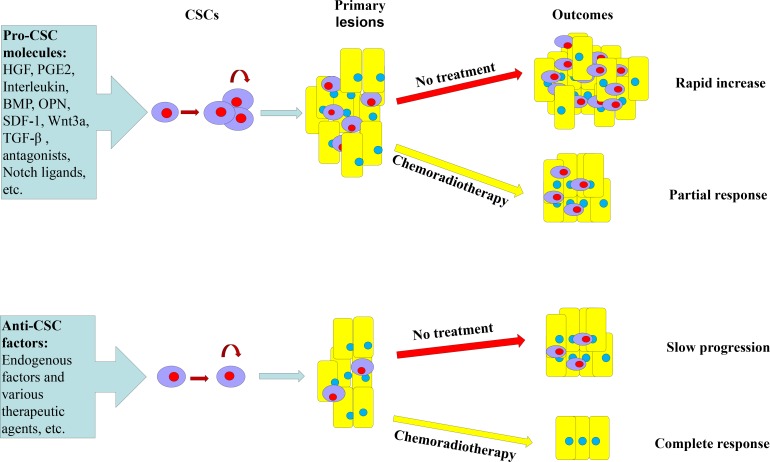
Microenvironmental molecules of colorectal CSCs Microenvironmental molecules of CSCs include two groups: Pro-cancer stem cells (Pro-CSC) molecules and anti-cancer stem cells (anti-CSC) molecules. The Pro-CSC molecules in the tumor microenvironment promote proliferation of CSC while anti-CSC factors promote CSC differentiation, lowering down CSC number. Chemoradiotherapy is scarcely effective in the presence of a Pro-CSC tumor microenvironment and therapeutic molecules that target CSC self-renewal or survival may kill CSCs. HGF, hepatocyte growth factor; PGE2, prostaglandin E2; OPN, osteopontin; SDF1, stromal-cell-derived factor 1; BMP, bone morphogenetic protein; IL, interleukin; and TGF-β, transforming growth factor beta.

Microenvironment of stem cells is a physiological environment to maintain their biological features; aberrations of microenvironment can induce normal stem cells into cancer stem cells. The CSC microenvironment is complex, in which there are cytokines and molecules that promote development of CSCs and there are also factors that prevent CSCs (Figure 2). The pro-CSC cytokines, i.e., hepatocyte growth factor (HGF), prostaglandin E2 (PGE2), bone morphogenetic protein (BMP) and interleukins produced by the tumor microenvironment, increase the CSC pool [58]. For example, MFG-E8 secreted by tumor-associated macrophages maintains self-renewal of colorectal CSCs through the STAT3/Sonic Hedgehog signaling pathway; knockdown of MFG-E8 in the tumor-associated macrophages significantly inhibited tumorigenicity of CSCs in immunodeficient mice [80]. Oppositely, anti-CSC molecules decrease CSC number by forcing sequential differentiation into precursors [18]. Traditional chemotherapeutic agents are less effective in the presence of a pro-tumor microenvironment, but therapeutic agents that target CSC self-renewal or survival may be active.

Distribution of cancer cells or cancer stem cells to specific organs is preferentially mediated by signals derived from the microenvironment, including oxygen gradient, chemokine receptors and cyclooxygenases [81–83]. Liver is a main target organ of colorectal cancer metastasis. In addition of the anatomical reason, chemokine receptor 4 (CXCR4) is an important factor involved in colorectal-liver metastases. CXCR4 is a specific receptor of stromal cell-derived factor 1 (SDF-1); the SDF-1 is highly expressed in the liver, which promotes the circulating CXCR4^+^ colorectal cancer cells to move into the liver [84]. TGF-β signaling pathway in hepatic stellate cells (HSCs) interacts with platelet-derived growth factor receptor alpha (PDGF-α), mediating proliferation and migration of CRC cells [85]. Thus HSCs with PDGF-α expression in the liver may form a microenvironment for colorectal-liver cancer metastasis. In addition, bone mesenchymal stem cells (MSCs) that migrate into the microenvironment of tumors *in situ* can differentiate into CRC-associated fibroblasts and promote cell invasion in the primary tumor site and metastasis to distant organs [86, 87]. Hence, CSCs have strong ability of migration and invasion, but the microenvironment provides specific biochemical factors for their metastasis to indicated tissues/organs with a specific microenvironment.

### Colorectal CSCs and cancer resistance to treatment

Traditional treatments for CRC include surgery, radiotherapy and chemotherapy. Resistance of CRC to radiotherapy and chemotherapy is a major cause of treatment failure and cancer death. Radiation or chemotherapeutic drugs may effectively kill more differentiated no-CSCs in a mass, but have limited effects on CSCs. In fact, colorectal CSCs are widely resistant to radio- and chemotherapy, being a key factor of treatment resistance and cancer recurrence [88–91]. The discovery of colorectal CSCs highlights the intratumoral heterogeneity [92].

Exact mechanisms of colorectal CSC resistance to radiation and chemotherapeutic drugs remain to be fully understood, but several potential descriptions have been discussed [93]. First, CSCs may not enter the proliferating cycle, but be quiescent in the G_0_ phase and thus be resistant to radio- and chemotherapy [94]. Second, CSCs have enhanced ability of DNA damage repair and thus are resistant to DNA-damaging radiation and agents [95]. Third, CSCs express high levels of anti-apoptotic proteins, including Bcl-2 family members and apoptotic inhibitors, and thus are resistant to apoptosis [96]. Finally, CSCs express high levels of ABC transporters and p-glycoproteins that pump chemotherapeutic drugs out of cells [97]. Recently, galectin-3 (Gal3) is identified as a novel protein mediating resistance to tumor necrosis factor-related apoptosis-inducing ligand (TRAIL) through blockade of intracellular trafficking of death receptors [98]. The Gal3 expression is associated with increased sphere-forming ability (SFA), ALDH activity and tumorigenesis, and thus Gal3-positive colorectal CSCs are resistant to chemotherapy regimens, such as FOLFOX (5-fluorouracil, oxaliplatin and leucovorin) and FOLFIRI (5-fluorouracil, irinotecan and leucovorin) [98].

PER3 is a protein that is negatively involved in colorectal CSC drug resistance. The PER3 expression in colorectal CSCs and drug resistant HCT-116 cells is low, and induction of PER3 leads to inhibition of self-renewal and sensitivity to 5-fluorouracil of colorectal CSCs [24]. This works through suppression of β-catenin expression. Aberrant activation of Wnt/β-catenin signaling promotes proliferation of colorectal CSCs by upregulation of c-Myc and cyclin D1 [99]. PER3 inhibits β-catenin expression and thus leads to inhibition of self-renewal and sensitivity of CSCs to 5-fluorouracil. In addition, interleukin-6 (IL-6) stimulates stemness of colorectal CSCs and induces resistance to 5-fluorouracil through activation of Notch-3 signaling pathway [100, 101]. Anti-IL-6 therapy can reduce the expression of Oct-4, Klf4, Bmi-1, Lgr5 and Notch-3 and increase cell sensitivity to chemotherapeutic drugs [102].

Radiotherapy is a major means of CRC treatment, which kills cancer cells through DNA damage by ionizing radiation and resultant reactive oxygen species (ROS). Colorectal CSCs demonstrate unique characteristics, such as upregulated anti-apoptotic proteins, enhanced DNA damage repair and dormancy/slow cell cycle kinetics, and thus are radioresistant [103, 104]. To date, the described mechanisms that cause radioresistance of colorectal CSCs include enhanced DNA damage repair, decreased cell cycle activity, high ROS inhibitors, resistance to apoptosis and activation of survival pathways, e.g., Notch, c-Jun N-terminal kinase and protein kinase C δ signaling pathways [89, 105–107]. Radiation resistance of colorectal CSCs is also related to adaptive response induced by ionizing radiation and microenvironmental changes, such as cytokines, nitric oxide and oxygen contents [94]. In addtion, radiation can induce non-stem cancer cells to obtain the phenotypes and function of CSCs, leading to resistance to radiotherapy [108]. The radiotherapy may also induce cancer cells to undergo EMT, thus resulting in gain of stemness and radioresistance [109, 110]. Inhibition of NF-κB signaling blocks radiation-induced stemness, and inactivation of Notch signaling inhibits EMT via downregulation of Snail, enhancing sensitivity to radiotherapy [89, 111].

### Colorectal CSCs and cancer recurrence

Traditional radiotherapy and chemotherapy can reduce the number of tumor cells and tumor volume, achieving remission of CRC on gross pathology, but the recurrence is high if the entire tumor is not surgically removed. Colorectal CSCs may be a key factor that promotes CRC recurrence. A principle limitation of current chemo-/radiotherapy is that it only eliminates more differentiated cancer cells, but not CSCs [112]. The CSCs tolerate or escape the destruction of chemotherapeutic agent and radiotherapy and survive. Due to their strong tumorigenicity, a small portion of survived CSCs in the quiescent status could re-enter into cell cycle for continuous proliferation by stimulation of appropriate signals in the microenvironment, leading to tumor recurrence. For instance, CD133^+^ colorectal cancer cells with high expression of ACBG2 and OCT-4 contribute to colorectal cancer recurrence [113].

## CONCLUSIONS

**Figure 3:**
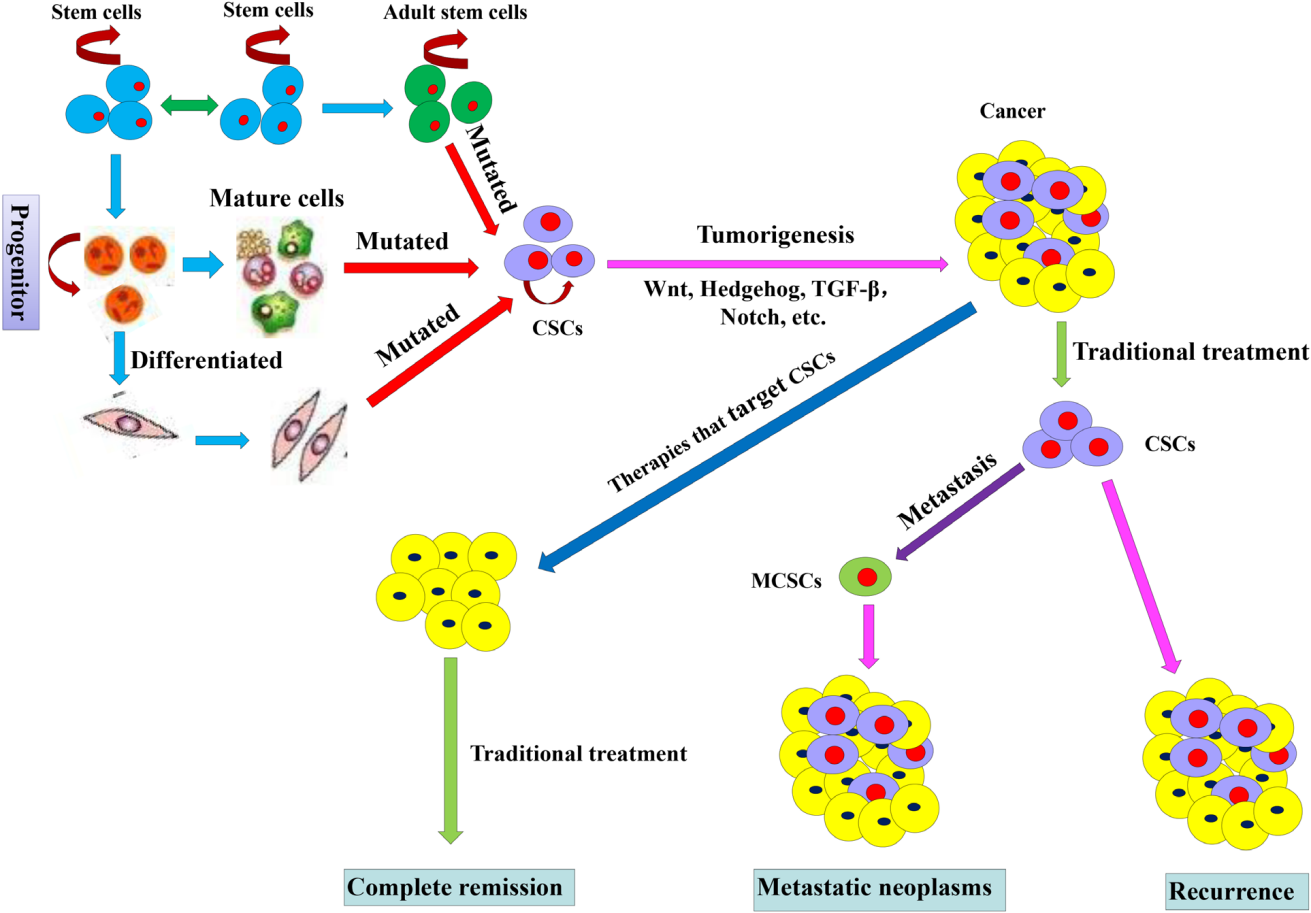
Origins and effects of colorectal CSCs on treatment CSCs with sustained self-renewal, persistent proliferation and tumor initiation originate from mutated adult stem cells, mature cells and differentiated cells. CSCs play a role of pro-tumorigenesis due to the active Wnt, Hedgehog, TGF- beta and Notch signaling pathways. Traditional treatment cannot kill all CSCs, and the survival CSCs lead to the cancer relapse and form migrating cancer stem cells (MCSCs) which form metastatic neoplasms in the distant organs. Primary cancer that accepts therapies targeting CSCs can achieve complete remission.

High mortality of CRC is ascribed to metastasis, treatment resistance and recurrence. Understanding of colorectal CSCs has opened a window to cure this cancer. Through dedication of cancer research scientists in the past decades, knowledge of colorectal CSCs has been enriched in terms of their origins, biomarkers, signaling transduction and biological functions in tumor metastasis, treatment resistance and relapse (Figure 3). This provides a novel platform for development of new treatment modes to overcome the shortage of traditional therapies and achieve the purpose of curing cancer. However, due to lack of highly specific markers and complex biological characteristics of colorectal CSCs, effectively targeted therapies remain to be explored. Continuing efforts of scientists are needed for accurate and effective treatment targeted to CSCs in CRC.

## Abbreviations

ABC transporter: ATP binding cassette transporter
ALDH1: aldehyde dehydrogenase isoform 1
BMP: bone morphogenetic protein
CSC: cancer stem cells
CCSC: circulating cancer stem cells
CRC: colorectal cancer
CRLM: colorectal-liver metastases
CRPM: colorectal-pulmonary metastases
CXCR4: chemokine receptor 4
EMT: epithelial mesenchymal transition
HGF: hepatocyte growth factor
HSC: hepatic stellate cells
ISC: intestinal stem cells
Lgr5: leucine-rich repeat-containing G protein-coupled receptor 5
MCSC: metastatic migrating cancer stem cells
MSC: mesenchymal stem cells
PGE2: prostaglandin E2
PDGF-α: platelet-derived growth factor receptor alpha
ROS: reactive oxygen species
SDF-1: stromal cell-derived factor
SFA: sphere-forming ability
SSCS: stationary cancer stem cells
TACSTD-1: tumor-associated calcium signal transducer-1
TCSC: tissue committed stem cells
TIC: tumor initiating cells
TRAIL: tumor necrosis factor-related apoptosis-inducing ligand
